# The response of sugar beet rhizosphere micro-ecological environment to continuous cropping

**DOI:** 10.3389/fmicb.2022.956785

**Published:** 2022-09-07

**Authors:** Rufei Cui, Gui Geng, Gang Wang, Piergiorgio Stevanato, Yinzhuang Dong, Tai Li, Lihua Yu, Yuguang Wang

**Affiliations:** ^1^National Sugar Crop Improvement Centre, College of Advanced Agriculture and Ecological Environment, Heilongjiang University, Harbin, China; ^2^Engineering Research Center of Agricultural Microbiology Technology, Ministry of Education, Heilongjiang University, Harbin, China; ^3^Heilongjiang Provincial Key Laboratory of Ecological Restoration and Resource Utilization for Cold Region, College of Life Science, Heilongjiang University, Harbin, China; ^4^Dipartimento di Agronomia, Animali, Alimenti, Risorse Naturali e Ambiente (DAFNAE), Università degli Studi di Padova, Padua, Italy

**Keywords:** sugar beet, continuous monocropping, compartments, microbiome, high throughput sequencing

## Abstract

Continuous cropping can lead to increased soil-borne diseases of sugar beet (*Beta vulgaris* L.), resulting in a reduction in its yield quality. However, our understanding of the influence of continuous cropping on sugar beet-associated microbial community is limited and their interactions remain unclear. Here, we described and analyzed microbial diversity (*N* = 30) from three sugar beet belowground compartments (bulk soil, rhizosphere soil, and beetroot) using 16S rRNA and ITS sequencing. The continuous cropping showed lower bacterial alpha diversity in three belowground compartments and higher fungal alpha diversity in roots compared to the non-continuous cropping. There were significant differences in fungal community composition between the two groups. Compared with non-continuous cropping, continuous cropping increased the relative abundance of potentially pathogenic fungi such as *Tausonia, Gilbellulopsis*, and *Fusarium*, but decreased the relative abundance of *Olpidium*. The fungal flora in the three compartments displayed different keystone taxa. Fungi were more closely related to environmental factors than bacteria. Overall, changes in microbial diversity and composition under continuous cropping were more pronounced in the fungal communities, and the results of the study could guide development strategies to mitigate continuous crop adversity.

## Introduction

Sugar beet (*Beta vulgaris* L.) is not only the raw material in the sugar industry but also an important global feed (Chhikara et al., 2019; Li et al., 2020). The planted area accounts for about one-third of the global sugar crop planting area and provides 16% of the world’s sugar production (Geng and Yang, 2015; de Oliveira et al., 2020). The cultivation area and yield of sugar beet are second only to sugar cane (Mall et al., 2021). A good soil environment is a prerequisite for high sugar yields in sugar beet, which is a taproot crop that should not be grown continuously (Geng et al., 2020; Huang et al., 2021). However, due to poor land use and cultivation practices, sugar beet is often grown in succession, resulting in poor plant growth, frequent pests and diseases, reduced sugar content, and lower yields (Huang et al., 2020).

Soil and crops harbor an extraordinarily rich diversity of microbiomes that can impact their health by influencing the nutrient element cycle and affecting soil fertility (Bai Y. et al., 2015; Zhu B. et al., 2020). Soil microorganisms play key roles in the cycle of soil nutrient elements and soil material metabolism but are sensitive to changes in environmental conditions (Bastida et al., 2021). They are affected by soil properties (Subhashini and Kumar, 2019). Most of these soil properties are associated with microbiomes, soil pH, and enzyme activity (Wang et al., 2020). Additionally, the root microbiome has been widely used as the best indicator of host plant fitness (Zhu et al., 2018). Notably, plant root-associated, bulk soil, and rhizosphere soil microbiomes have caught widespread attention due to their crucial roles in host growth and development (Fitzpatrick et al., 2018).

Black soils play a key role in the crucial soil resources of Northeast China and are one of the most important factors in lasting national food security (Liu Z. et al., 2020). The intensification of sugar beet monoculture is happening on a wide scale in these areas due to limitations in cultivated land, economic benefits, and a requirement for enhancing regional agricultural industrialization (Samadi et al., 2008). Recent studies have shown that long-term continuous cropping affects the plant root-associated, bulk soil, and rhizospheric soil microbial structure (Miao et al., 2016; Bracho-Mujica et al., 2019; Liu H. et al., 2020; Yuan M. et al., 2021). In contrast, such variations further contribute to the degree of long-term continuous cropping obstacles (Sun et al., 2017). Thus, the healthy and stable microbial community structure in the microecology may be essential for maintaining stable crop yields and relieving continuous cropping obstacles (Gao et al., 2019). It has recently been found that continuous cropping can lead to changes in soil chemical properties (Perez-Brandan et al., 2014), alterations in soil enzyme activity, soil-borne pathogen accumulation (Xiong et al., 2015), enrichment of allelochemical substances, and soil microbial community changes (Bai L. et al., 2015). Therefore, understanding how plant root-associated, bulk soil, and rhizosphere soil microbiomes respond to agricultural management measures and crop physiological conditions is of great significance to agricultural production (Chen et al., 2019).

The effects of continuous cropping obstacles on soil microorganisms and plant growth have been studied (Huang et al., 2020, 2021). However, a comprehensive study of the diversity of soil and root microbial communities during the growth of sugar beet seedlings in continuous cropping soil has not yet been reported. In this study, we performed pot experiments to examine the microbial community composition and functions of sugar beet bulk soil, rhizosphere soil, and seedling roots in continuous cropping and non-continuous cropping systems using high-throughput sequencing technology. The main objectives of the study were (1) to reveal the effect of continuous cropping on the composition and diversity of bacterial and fungal communities in three belowground compartments (bulk soil, rhizosphere soil, and beetroot); (2) to evaluate the correlation of microbial changes with soil characteristics; and (3) to predict changes in the ecological functions of microbes in sugar beet continuous cropping system. This study aimed to provide a theoretical basis for mitigating succession barriers in sugar beet and to provide important guidance for developing improved agricultural regulatory strategies.

## Materials and methods

### Plant material and experimental design

Seeds of sugar beet (KWS1176 from KWS Company of Germany) were selected as plant materials in this experiment. KWS1176 is a pelletized seed which is film-coated and wrapped in a thin layer containing thiram to kill pathogens on the seeds after disinfection. In our previous studies, it was found to exhibit strong resistance to adversity and is more widely used in Northeast China. The soil was sourced from the black soil area in Hulan District, Harbin City, Heilongjiang Province (latitude and longitude: 46°00′14′′ E, 126°38′49′′ N). Soil samples included soils from a maize-beet rotation (previous crop was maize) and soils from 3 years of continuous sugar beet crop (2-year recrop soils), both collected from adjacent plots with similar agricultural management practices. We sowed the beet pelleted seeds after washing off the coating with sterile water. The seeds were sown in a plastic pot containing 0.7 kg of soil. In this experiment, each pot was sown with six seeds and was irrigated with 50 mL of Hoagland nutrient solutions every 10 days. The samples were distributed as follows: non-continuous cropping group and continuous cropping group. To ensure the normal growth of plants, only one seedling was selected in each pot after 5 days of planting. All pots were incubated in a laboratory with the following photoperiod: day (lights on) 7 am–9 pm, 24°C; night (lights off), 19°C.

### Plant and soil sampling

Harvesting was carried out on the 30th day. Beetroots, rhizosphere soils, and bulk soils were processed as previously described (Bulgarelli et al., 2012). Each treatment group was randomly selected from uniformly growing sugar beet, and five replicates of each treatment were recorded as one replicate by taking three pots of samples and mixing them. Beet plants were manually collected from the plastic pots and bulk soil aggregates were separated by shaking plant roots. The bulk soil samples were sampled from the pots and passed through the sterile 2-mm sieve. The bulk soil was divided into two parts for subsequent analysis. One portion was air-dried for soil properties and soil enzyme activities, and the other portion was frozen in liquid nitrogen and stored at −80°C for the sequencing of microbes. To standardize the sampling, we used a sterile scalpel to dissect the roots under the beet seedling stems to maximize repeatability. Beetroots were pooled into a sterile 50 mL tube containing 25 mL of sterile Silwet L-77 amended phosphate-buffered saline solution (PBS) (per liter: 7 mM Na_2_HPO_4_, 3 mM NaH_2_PO_4_, 200 μL Silwet L-77, and pH 7.0) and were vigorously mixed with a vortex for 15 s to detach from the root surfaces to produce the rhizosphere soil. Then samples were subjected to centrifugation at 3,200 g for 15 min to get the rhizosphere soil. For beetroot DNA extraction, roots were transferred to a new 50 mL sterile tube containing 25 mL of PBS, and vortexed. Repeat the steps until the phosphate buffer in the centrifuge tube was no longer turbid. Then, the root was transferred into another sterile tube and sonicated for 10 cycles, consisting of 30 s bursts and rests of 30 s. The purpose of this step was to remove the microorganisms attached and further clean the outer surface of the roots. Finally, the roots were transferred to a fresh volume of 25 mL of PBS and the sonicated roots were defined as beetroot. The beetroot samples and rhizosphere soil were then stored at −80°C until DNA extraction. Through the use of scanning electron microscopy and microbial culture techniques, Xiao et al. (2017) confirmed that the microorganisms on the root surface could be removed by the above steps.

### Determination of soil properties and plant indicators

Five plants were randomly selected for plant height, root fresh weight, and dry weight. Five sugar beet plants of uniform growth were randomly selected from each treatment group and washed with deionized water for measuring root vigor. The root vitality of the sugar beet plants was measured according to the alpha naphthylamine method (Ramasamy et al., 1997). Root morphology and root area (five plants) were determined by WINRHIZO software (Regent Instruments Inc., Quebec, QC, Canada). The air dried bulk soil samples were passed through a 2-mm sieve as described by Bao (2005). Air-dried soils were used to determine soil physicochemical properties and soil enzyme activity (pH, EC, AN, AK, AP, sucrase, acid phosphatase, CAT, and urease). Specifically, the hydrogen potential (pH) and electrical conductivity (EC) of the soil were measured with a potentiometer after shaking for 30 min at the soil to distilled water ratio of 1:2.5. Soil available nitrogen (AN) was measured by changing available nitrogen into ammonia. Soil available phosphorus (AP) was determined by leaching with sodium bicarbonate solution and molybdenum-antimony colorimetric method. Soil available potassium (AK, extracted in ammonium acetate) was determined by the flame photometer. Soil catalase activity (CAT) was measured according to KMnO_4_ titration. Soil urease was based on urea and the enzyme activity was determined based on the enzymatic product ammonia interacting with phenol-sodium hypochlorite to produce blue indophenol. Soil sucrase content was determined by a colorimetric method using 3,5-dinitrosalicylic colorimetry and the amount of reducing sugars was used to express the enzyme activity. Soil acid phosphatase activity was determined by a colorimetric method using sodium benzene phosphate to express enzyme activity as phenol content. All bulk soil samples were assayed in five replicates, and inorganic controls were required for each treatment for the soil enzyme assay.

### DNA extraction and amplicon sequencing

The non-continuous cropping sugar beet bulk soil (Sn), its rhizosphere soil (Rn), its beetroot (Bn), and continuous cropping samples (Sc, Rc, and Bc) were extracted as research materials. DNA was extracted from bulk soil, rhizosphere soil, and root samples (0.5 g per sample) by the E.Z.N.A^®^. Soil DNA Kit (Omega Bio-tek, Norcross, GA, United States). DNA quality and integrity were examined using 1% agarose gel electrophoresis. 16S rRNA genes were selected for the V3–V4 region and fungal ITS amplification was selected for the ITS1–ITS2 region (Huang et al., 2020). This 16S and ITS high throughput sequencing was performed by BIOZERON Ltd. (Shanghai, China).

### Processing of sequencing data

The MiSeq platform PE250 (Illumina, Inc., CA, United States) was used for double-end sequencing and the PANDAseq software was used for splicing to obtain long reads with highly variable regions. QIIME was used to analyze the raw sequence data, and the 250 bp reads were truncated, receiving a low-quality score (≤20), and those shorter than 50 bp were discarded (Caporaso et al., 2010). Removal of chimeras using UCHIME software (Edgar et al., 2011). OTU clustering based on 97% sequence similarity using the UPARSE plug-in (Edgar, 2010). Species annotation of OTU representative sequences against the Greengenes database was performed using the RDP classifier (80% confidence interval) (Wang et al., 2007). OTUs with annotated results for chloroplasts and mitochondria or OTUs with only one sequence (singletons) were removed. Given the variation in sequencing depth between samples, the OTUs were normalized for subsequent analysis using the least square method.

### Statistical analyses

The rarefaction was calculated using the software Mothur (Schloss et al., 2009) and conducted to reveal the alpha diversity, including the Chao1 and Richness indices (Schloss et al., 2011). To test the statistical significance of the structural similarity between communities with different sampling treatments, UniFrac-based hierarchical clustering (Lozupone et al., 2011) was performed using the community ecology package (Wang et al., 2012). Displacement multivariate analysis of variance (PERMANOVA) and non-metric multidimensional scaling analysis (NMDS) were used to examine and visualize differences in the structure of the bacterial and fungal communities in the three compartments, respectively. To identify biomarkers of important microbial taxa, LEfSe (linear discriminant analysis effect size) analysis was performed as previously reported (Segata et al., 2011). The relationship between changes and dissimilarities was analyzed by the Kruskal–Wallis sum-rank test, and the threshold was set at 0.05. For LDA analysis, the threshold for the size effect of each OTU was set at the threshold for abundant taxa (Ijaz et al., 2018). To further evaluate the relationship between microbial community composition and nine factors of physicochemical properties, the spearman rank correlation and mantel tests were visualized using the “ggcor” software package (Yuan M. M. et al., 2021). Functional annotation of 16S rRNA bacterial gene sequences from the SILVA database was performed using the Tax4Fun software package and the FAPROTAX package. Analysis of variance, Tukey’s test, and Duncan’s test were performed using IBM SPSS 19.0 software. Box line plots were drawn using PRISM (Version 7.0) software. The rest of our analyses were performed with the aid of R software (Version 4.0.0) and the BIOZERON cloud platform^1^.

## Results

### Soil properties and plant growth indicators

While the soil pH and AK were decreased, EC, AP, and AN were increased in the continuous cropping group compared to that of the non-continuous cropping group (Table 1). In addition, the continuous cropping group exhibited higher Catalase and urease activities, while sucrase and acid phosphatase activities were higher in the non-continuous cropping group (Table 1). We found that the plant height (Height), fresh weight (FW), root dry (DW), root vitality (RA), and root surface area (RSA) in the site with the continuous cropping group were significantly lower than the group with the non-continuous cropping group (Table 2 and Supplementary Figure 1).

**TABLE 1:** Physico-chemical properties and soil enzyme activity of bulk soils in different treatment groups.

Group	pH	AN (mg/kg)	AP (mg/kg)	AK (mg/kg)	EC (Ms/cm)	Catalase (mg/g)	Urease (mg/g)	Phosphatase (mg/g)	Sucrase (mg/g)
Sc	6.856 ± 0.009b	59.80 ± 0.071a	194.818 ± 0.029a	240.40 ± 1.042b	497.0 ± 1.581a	1.043 ± 0.023b	0.407 ± 0.003b	0.677 ± 0.008a	58.571 ± 0.701a
Sn	7.440 ± 0.012a	54.42 ± 0.170b	170.451 ± 0.985b	243.62 ± 1.121a	425.8 ± 0.837b	1.170 ± 0.015a	0.447 ± 0.016a	0.377 ± 0.020b	48.148 ± 0.806b

pH, AP, AK, EC represent available nitrogen, pH, available phosphorus, total potassium and available potassium. Sc, continuous cropping bulk soil; Sn, non-continuous cropping bulk soil. Values are means ± standard deviation (*n* = 5), followed by the same letter for a given factor which are not significantly different (*P* < 0.05; Wilcoxon test).

**TABLE 2:** Plant indicators for different groups.

Group	Height (cm)	FW (g)	DW (g)	RA (μg/g*h)	RSA (cm^2^)
Bc	10.967 ± 0.503b	3.00 ± 2.176b	0.271 ± 0.012b	197.934 ± 6.885b	58.080 ± 3.323b
Bn	14.200 ± 0.047a	7.97 ± 0.818a	1.192 ± 0.059a	262.911 ± 9.108a	121.747 ± 23.895a

Bc, continuous cropping sugar beetroot; Bn, non-continuous cropping sugar beetroot. Values are means ± standard deviation (*n* = 5), followed by the same letter for a given factor which are not significantly different (*P* < 0.05; Wilcoxon test).

### Alpha diversity of the microbial community

**FIGURE 1:**
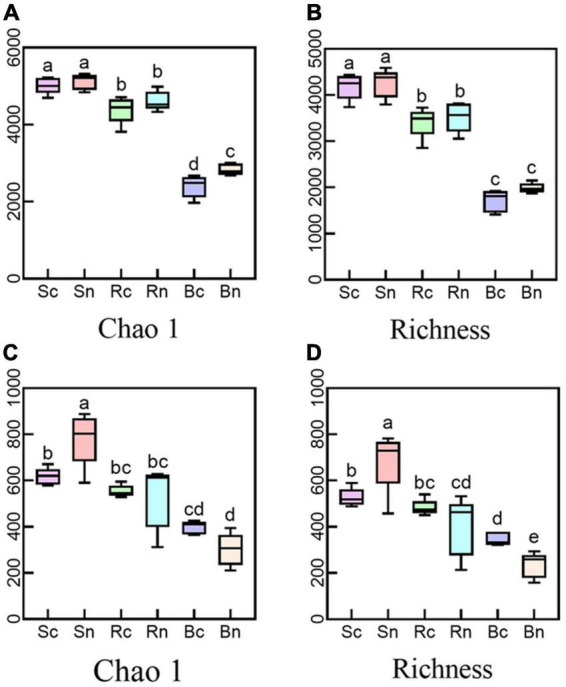
Alpha diversity of the bacterial and fungal communities. Panels **(A,B)** representing alpha diversity of the bacterial community; **(C,D)** representing alpha diversity of the fungal community. Different letters (*P* < 0.05) were considered to have Significances on the top. Sc, continuous cropping bulk soil; Sn, non-continuous cropping bulk soil; Rc, continuous cropping rhizosphere soil; Rn, non-continuous cropping rhizosphere soil; Bc, continuous cropping sugar beet root; Bn, non-continuous cropping sugar beetroot.

We evaluated the alpha diversity of different compartments using the Chao1 and Richness indices to measure microbial community diversity and richness, respectively (Figure 1 and Supplementary Table 1). The continuous cropping group decreased the bacterial richness index (Chao1 and Richness) in three belowground compartments compared to the non-continuous crop group. However, the continuous cropping group had different effects on the fungal richness indices of the three underground compartments. The continuous cropping group significantly decreased the fungal diversity of the bulk soil and increased the fungal diversity of the beetroot compared to the non-consecutive cropping group. The results of the two-way ANOVA (Supplementary Table 2) showed that compartment had a significant effect on alpha-diversity (*P* < 0.01). This indicated that continuous cropping can affect the alpha diversity in plants and soils.

### Beta diversity of the microbial community

**FIGURE 2:**
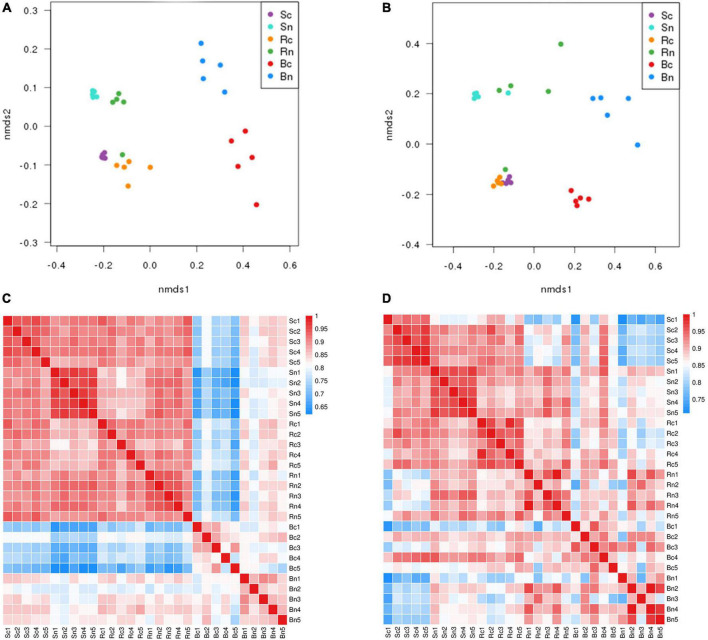
Spearman clustering heatmap and Unweighted UniFrac NMDS of the bacterial and fungal communities. Panels **(A,C)** representing clustering heatmap and NMDS of the bacterial community; **(B,D)** representing clustering heatmap and NMDS of the fungal community. The color code indicated relative abundance, ranging from blue (low correlation) to red (high correlation). Sc, continuous cropping bulk soil; Sn, non-continuous cropping bulk soil; Rc, continuous cropping rhizosphere soil; Rn, non-continuous cropping rhizosphere soil; Bc, continuous cropping sugar beetroot; Bn, non-continuous cropping sugar beetroot.

The NMDS with the unweighted UniFrac algorithm demonstrated that the bacterial and fungal communities from the different compartment samples were clustered separately (Figures 2A,B). It showed that these samples under continuous cropping group and non-continuous cropping group formed distinct clusters in the plotted ordination space. The bulk soil and rhizosphere soil were clustered together, while they were significantly separated from the beetroot. A heat map of the Beta diversity index was constructed (Figures 2C,D). The results revealed that the bulk soil shared the highest level of correlation with rhizosphere soil. It suggested that microbial community composition varies considerably across compartments. Moreover, the soil and beetroot microbial communities differed considerably between the two treatment groups.

### Microbial community structure variations

This study also showed the composition of bacterial and fungal species from phylum to genus level in beetroot, rhizosphere soil, and bulk soil samples subjected to the different types of soil treatment alternatives. The proportional abundance of dominant taxa changed under different cropping systems and compartment conditions.

**FIGURE 3:**
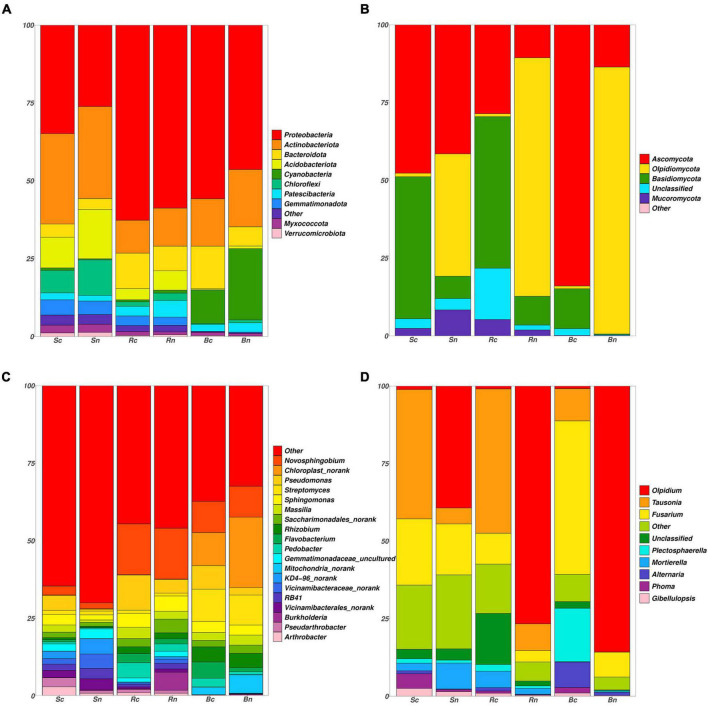
Phylum and genus level taxonomic differences in bacterial and fungal communities. Distribution of the most abundant bacterial **(A)** and fungal **(B)** phyla. The area on the Outer left ring represents the contribution of each phylum in each sample. Distribution of the most abundant bacterial **(C)** and fungal **(D)** genera. Sc, continuous cropping bulk soil; Sn, non-continuous cropping bulk soil; Rc, continuous cropping rhizosphere soil; Rn, non-continuous cropping rhizosphere soil; Bc, continuous cropping sugar beetroot; Bn, non-continuous cropping sugar beetroot.

The operational taxonomic units (OTUs) of the different compartments of bacterial communities belonged to 39 phyla, 113 classes, 259 orders, 413 families, and 846 genera. The top 10 bacterial phyla in terms of compartment abundance included Proteobacteria, Actinobacteria, Bacteroidota, Acidobacteriota, Cyanobacteria, Chloroflexi, Patescibacteria, Gemmatimonadetes, Myxococcota, and Verrucomicrobiota (Figure 3A). The total of these phyla accounted for more than 98.2% of the bacterial sequences. Proteobacteria and Actinobacteria accounted for 66% of the bacterial community and were the two largest phyla. The relative abundance of Proteobacteria increased in the continuous cropping, respectively, compared to levels in the non-continuous cropping, and the abundance of rhizosphere soil and beetroot was greater than that of bulk soil (Figure 3C). Furthermore, the abundance of Actinobacteriota decreased. *Pseudomonas, Flavobacterium*, and *Pedobacter* were significantly higher in abundance in the continuous cropping samples. In contrast to bulk soil, the rhizosphere soil and beetroot harbored a higher portion of *Novosphingobium*.

The fungal OTUs belonged to 7 phyla, 25 orders, 71 families, 163 families, and 363 genera in the high-throughput sequencing results. The four phyla were Ascomycota, Olpidiomycota, Basidiomycota, and Mucoromycota (Figure 3B). Ascomycota was dominant in the continuous cropping group, especially continuous cropping beetroot (84.0%), and Olpidiomycota was dominated by non-continuous cropping beetroot (85.8%) and rhizosphere soil (76.7%). The relative abundance of *Tausonia, Fusarium*, and *Gilbellulopsis* increased in the continuous cropping, respectively, compared to levels in the non-continuous cropping. Moreover, the *Tausonia* abundance of rhizosphere and bulk soil was greater than that of beetroot, and the *Fusarium* abundance of beetroot is greater than that of other treatment groups (Figure 3D). Furthermore, the continuous cropping group *Olpidium* was reduced and had the lowest abundance compared to the non-continuous cropping group.

**FIGURE 4:**
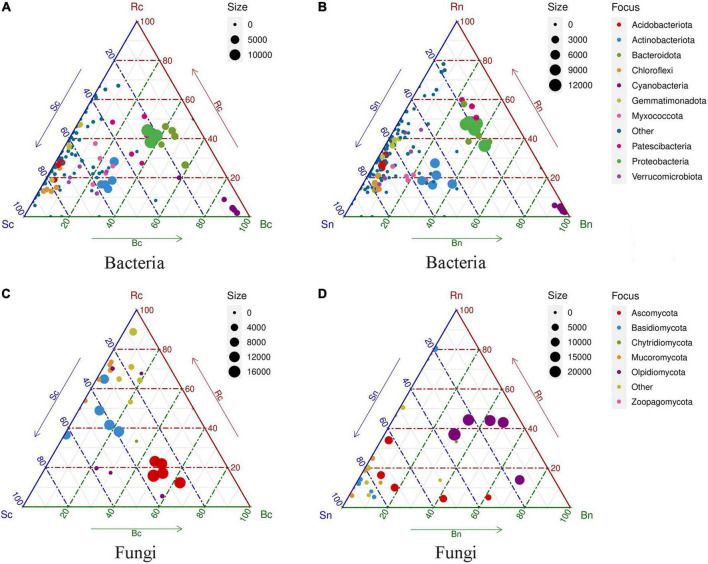
Ternary plots indicating compartment specificity of phyla from bacterial and fungal communities. Each color circle represents a phylum level. The dimension of each circle represents its weighted average. The position of each circle is decided by the contribution to the three compartments (bulk, rhizosphere, and beetroot) to the total weighted average. The dotted latticework of each triangle indicates 20% increments of contribution. Panels **(A,B)** representing ternary plots of the bacterial community of two treatment groups; **(C,D)** representing ternary plots of the fungal community of two treatment groups. Sc, continuous cropping bulk soil; Sn, non-continuous cropping bulk soil; Rc, continuous cropping rhizosphere soil; Rn, non-continuous cropping rhizosphere soil; Bc, continuous cropping sugar beetroot; Bn, non-continuous cropping sugar beetroot.

In addition, ternary plots showed that many phyla were present in similar proportions in the three compartments (bulk soil, rhizosphere soil, and beetroot), but that some were comparatively more abundant at a specific position (Figure 4). We found that a proportion of bacterial phyla were significantly enriched in beetroots, while bulk and rhizosphere soils shared the majority of bacterial phyla (Figure 4A). The ternary plots showed that the dominant bacterial phylum was similarly distributed in the different compartments, while the fungal phylum differed more microbially at the level of the fungi. The specific fungal phylum in the beetroots of the continuous cropping group was Ascomycota, whereas the specific fungal phylum in the non-continuous cropping group was Olpidiomycota in both rhizosphere soil and beetroots (Figure 4B).

### Discovery of biomarkers in microbial communities

**FIGURE 5:**
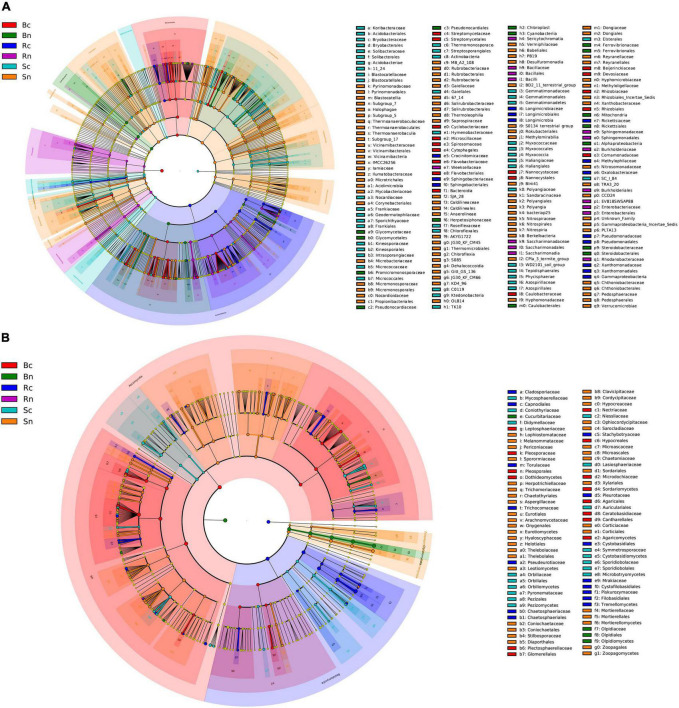
Cladogram obtained from LEfSe analysis showing changes in different abundances of bacterial **(A)** and fungal **(B)** community at different taxonomic levels. The circles radiating from inside to outside represent the taxonomic level from phylum to genus. The different color nodes in the branches represent the microbial groups that play an important role in the color group. The species names represented by the English letters in Figure are displayed in the legend on the right. Sc, continuous cropping bulk soil; Sn, non-continuous cropping bulk soil; Rc, continuous cropping rhizosphere soil; Rn, non-continuous cropping rhizosphere soil; Bc, continuous cropping sugar beetroot; Bn, non-continuous cropping sugar beetroot.

Differences in compartment microorganism community were assessed using linear discriminant analysis effect size (LEfSe) analysis at a linear discriminate analysis (LDA) threshold of 3 (Figure 5). Across the bacterial community, there were more species of Actinobacteria, Acidobacteria, Bacteroides, Proteobacteria, and there were species differences between the six test groups (Figure 5A).

For root microbial communities, continuous biomarkers included *Flavobacterium* (its phylum to genus), *Streptomyces* (its order to genus), *Rhizobium* (its order to genus), Comamonadaceae (family), *Pseudoxanthomonas* (genus), and *Stenotrophomonas* (genus), while the non-continuous cropping biomarkers mainly included *Glycomyces* (its phylum and order), *Lechevalieria* (its order to genus), and *Steroidobacter* (its order to genus). For the rhizosphere microbial community, continuous cropping rhizosphere soil biomarkers included *Massilia* (its order, family and genus), *Pseudomonas* (its order to genus), *Pedobacter* (by order to genus), Xanthomonadaceae (its order and family) and Proteobacteria (family), while the non-continuous cropping rhizosphere soil biomarkers mainly included *Novosphingobium* (its order to genus) and *Burkholderia* (its order to genus). In the bulk soil microbial community, Gemmatimonadaceae (its phylum to family), Intrasporangiaceae (its phylum to family), Frankiales (order), Gaiellales (order), Micrococcaceae (family), *Arthrobacter* (genus), and *Pseudarthrobacter* (genus) were significantly enriched in the continuous cropping group, while *RB41* (its phylum to genus), Vicinamibacteraceae (its order to family), Nocardioidaceae (its order to family), Solirubrobacterales (its order and phylum) Microtrichales (order), MB_A2_108 (order), Chloroflexia (its phylum and order), and KD4_96 (order) were significantly enriched in non-continuous cropping group.

Among the fungal communities, Ascomycota and Basidiomycota species were more numerous, and there were species differences between the five test groups (Figure 5B). For the beetroot microbial community, the phylum level of continuous cropping beetroot biomarkers included Ascomycota and Basidiomycota, while the genus level included *Alternaria, Plectosphaerella, Dactylonectria*, and *Fusarium*, while *Olpidiomycota* (from phyla to genus) was significantly enriched in non-continuous cropping beetroot. For the rhizosphere soil microbial community, continuous cropping rhizosphere soil biomarkers included *Trichocladium* and *Tausonia* (from phylum to genus). In the bulk soil microbial community, *Phoma* (its family and genus), *Pseudombrophila* (by order to genus), and *Gibellulopsis* (genus) were significantly enriched in continuous cropping bulk soil, while Chaetomiaceae (its order and family), Helotiales (its class and order), and *Mortierella* (by order to genus) were significantly enriched in non-continuous cropping bulk soil.

### Environmental drivers of compartments microbial community composition

**FIGURE 6:**
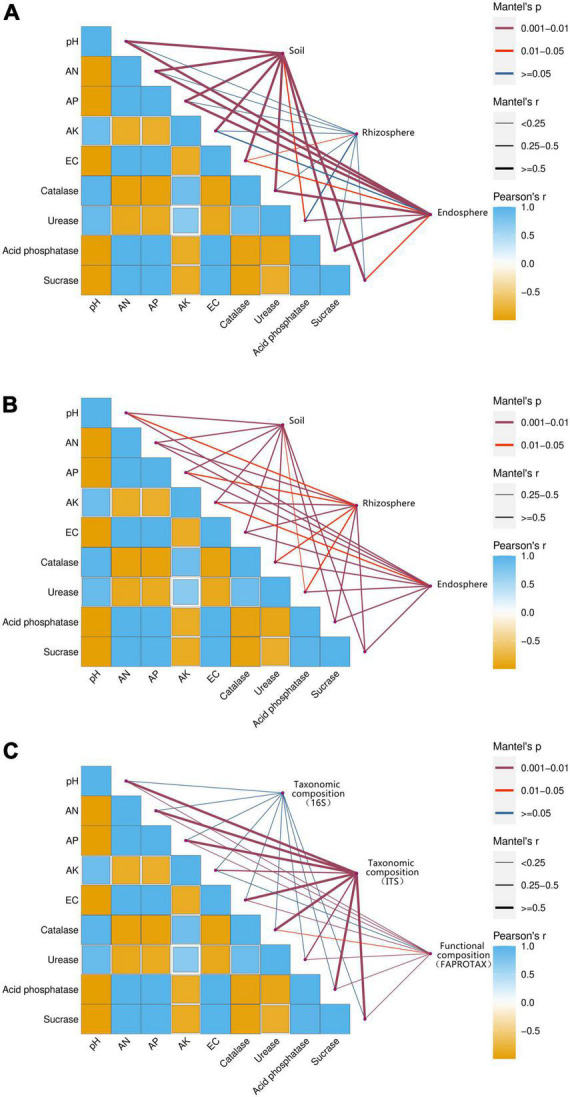
Environmental drivers of compartments microbial community composition. Compartments (based on bulk soil, rhizosphere soil, and beetroot) microbial community composition of bacteria **(A)** and fungus **(B)** related to each environmental factor by partial Mantel tests. Taxonomic (based on 16S and ITS) and functional (based on FAPROTAX annotation) microbial community composition **(C)** related to each environmental factor by partial Mantel tests. The color gradient indicates Spearman’s correlation coefficients, with more positive values (dark blue) indicating stronger positive correlations, and more negative values (dark yellow) indicating stronger negative correlations. The edge widths correspond to Mantel’s *r* statistic for the correlations.

The environmental drivers of changes in the microbiome of three belowground compartments were explored by calculating correlations between the microbial community composition and soil properties (Figure 6). To conclude which environmental factors caused changes in the composition of the microbial community in the compartments, we correlated differences in functional community composition with soil properties employing distance correction. Soil pH, catalase, urease, and AK were significantly and negatively correlated with AN, AP, EC, acid phosphatase, and sucrase. As shown in Figure 6, the environmental factors were highly correlated with both bulk soil and beetroot of the bacterial (Figure 6A) and fungal (Figure 6B) microbiomes (*P* < 0.05). The fungal microbiome of rhizosphere soil was significantly related to environmental factors (*P* < 0.05). However, the bacterial rhizosphere soil microbiome only had a significant correlation with EC. This suggested the diversity and composition of the rhizosphere fungal community were more closely related to environmental factors than to bacteria. Besides, environmental factors were significantly related to both the functional (based on FAPROTAX annotation) and fungal taxonomic composition of the compartments’ microbiome. The bacterial taxonomic composition had no significant correlation with the soil environmental factors (*P* > 0.05).

### Functions of bacterial communities

**FIGURE 7:**
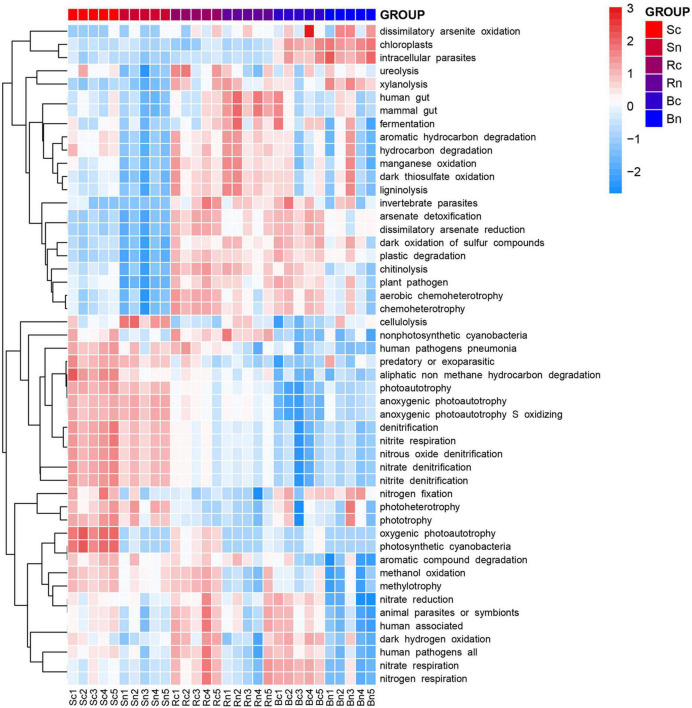
Heatmap of the predicted functional profile for the microbial communities at OUT level based on the Functional Annotation of Prokaryotic Taxa (FAPROTAX 1.1) database. The color code indicated relative abundance, ranging from blue (negative correlation) to red (positive correlation). Sc, continuous cropping bulk soil; Sn, non-continuous cropping bulk soil; Rc, continuous cropping rhizosphere soil; Rn, non-continuous cropping rhizosphere soil; Bc, continuous cropping sugar beetroot; Bn, non-continuous cropping sugar beetroot.

In the FAPROTAX database of microbial ecological function predictions, the annotated OTU was assigned to 61 predicted functional groups. Nevertheless, in the Kruskal–Wallis test, only 50 groups showed significant differences between the six compartments (*P* < 0.05). Therefore, they were plotted as a functional heatmap (Figure 7). Among these function predictions, nitrogen (10 groups), carbon (6 groups), sulfur (3 groups), and manganese (1 group) were involved in the geochemical cycle. The N cycle-related functions showed different performances in the three compartments, and the N cycle-related functions were basically positively correlated with the bulk soil compartment, such as denitrification, nitrate denitrification, nitrate respiration, and nitrous oxide denitrification. Continuous cropping of bulk soil enhanced the soil’s functional advantages of phototrophy, photosynthetic cyanobacteria, oxygenic photoautotrophy, and aliphatic non-methane hydrocarbon degradation. These functions, including dark oxidation of sulfur compounds, nitrogen respiration, nitrate respiration, and human pathogens, were significantly (*P* < 0.05) enhanced in the rhizosphere soil and root zone of the continuous cropping area compared to the non-continuous cropping area.

The first reference pathway that contained genes in the first five of the KEGG pathway analysis was metabolic, environmental information processing, genetic information processing, human diseases, and cellular processes. The number of metabolic pathway genes with the highest content was about 25 times higher than that of the second residual genes (Supplementary Figure 2A). The metabolic pathways of the second reference pathway containing the first five genes in KEGG pathway analysis were global and overview maps; carbohydrate metabolism, amino acid metabolism; energy metabolism; and membrane transport (Supplementary Figure 2B). The metabolic pathways in which the third reference pathway contained the first five genes in KEGG pathway analysis are metabolic pathways, biosynthesis of secondary metabolites, microbial metabolism in diverse environments, biosynthesis of antibiotics, and carbon metabolism (Supplementary Figure 2C).

## Discussion

Compartment microorganisms are affected by environmental factors and play an important role in the plant host and soil (Yu et al., 2021). This experiment performed high-throughput sequencing on the bulk soil, rhizosphere soil, and beetroot of continuous cropping and non-continuous cropping of sugar beet. The alpha diversity of bacteria and fungi decreases from bulk soil to rhizosphere soil to roots. In general, three compartments’ bacterial diversity decreased and the root fungal diversity increased in the continuous cropping group. The structural characteristics of the microbiota in the three compartments (bulk soil, rhizosphere soil, and beetroot) indicated different key taxa, suggesting that key microorganisms were attracted to the appropriate location (Hernandez-Alvarez et al., 2022). The results of the study of sugar beet microflora showed that Actinomycetes and Proteobacteria are their dominant bacterial phyla. Among the Proteobacteria phylum, *Pseudomonas* resulted as the most abundant genus across sugar beet samples, in agreement with recent studies (Bertoldo et al., 2021; Della Lucia et al., 2021). The relative abundance of the dominant fungal genera, *Tausonia* and *Fusarium* in continuous cropping of sugar beet is higher than that in rotation. It is speculated that these fungi may be closely related to the growth and development of continuous cropping sugar beet. In addition, *Fusarium* may be a pathogen that causes diseases in many plants (Li et al., 2014).

In the three compartments, the bacterial community levels of continuous cropping and non-continuous cropping groups existed in similar proportions, but the fungal communities were quite different. In the bacterial community, most of the bacterial phyla are enriched in bulk soil and rhizosphere soil. The roots, on the other hand, were enriched in Verrucomicrobia. In the fungal community, the dominant phyla of the two treatments are quite different. Ascomycetes were enriched in the three compartments of the continuous cropping group, while Olpidicota was abundant in the rhizosphere soil and root fungi of the non-continuous cropping group. The analysis of the LEFSE diagram showed that the specific genera of each compartment were different. This proved that the colonization of certain fungi may be related to host plants and treatment groups (Zhu L. et al., 2020). Some relatively stable symbiotic relationships may have formed between the three. It can be seen from the genus histogram that the key genus (*Olpidium, Fusarium*, and *Plectosphaerella*) present a gradient change from outside to inside in the three compartments. Because of the specific selection of roots, the colonization of fungal communities on roots is more selective. Therefore, from the outside to the inside of the three compartments, the structural characteristics of the fungal community showed different changes.

Microorganisms in plant compartments are the comprehensive performance of the environment and plants, which can effectively reflect a certain trend in their changes (Fierer, 2017). In the continuous cropping group, the indexes of soil pH, catalase, urease, AK, and AN decreased, while the indexes of AP, EC, acid phosphatase, and sucrase increased. This is consistent with the changing trend of environmental factors in other plant continuous cropping studies (Lei et al., 2020; Wang et al., 2020; Zhu B. et al., 2020). Continuous cropping will cause a drop in soil pH and changes in soil nutrients and enzyme activities (Wu et al., 2016; Arafat et al., 2019; Chen et al., 2020). The diversity and composition of the rhizosphere fungal community were more closely related to environmental factors than to bacteria. Environmental factors had significant correlation for the function of the community and fungal taxonomic composition. Taken together, the analysis showed that environmental factors had a significant relevance for the fungal community and functional composition but not with the bacterial community. Therefore, the response of the microbial community to environmental factors may have an impact on the abundance and composition of plant fungus-associated microorganisms, thereby affecting the plant host.

Previous studies have shown that nitrogen and carbon metabolism will affect or be affected by the microbial communities’ structure (Liao et al., 2021). This study compared the effects of continuous cropping treatment and non-continuous cropping treatment on the function (FAPROTAX) of the sugar beet compartments bacterial community. According to the FAPROTAX, the continuous cropping treatment reduced the abundance of certain nitrogen metabolisms and increased the abundance of carbon metabolisms. According to reports, amino acid metabolism promotes the growth and activity of microorganisms by providing them with more carbon, nitrogen, and energy (Liang et al., 2020). Tax4Fun function prediction analysis can be separated into three levels (level 1, level 2, and level 3). The first level was the largest and presents a gradual inclusion trend. Metabolism in the first class was dominant, while global and overview maps, carbohydrate metabolism, and amino acid metabolism were dominant in the second level. In the third class, the metabolic pathways were dominant. However, there was no significant difference in metabolism in different groups (*p* > 0.05). This may be due to the large base of soil microbial species and numbers, where changes in the function of a few specific bacteria are not reflected in the overall data. There is also a specific group of terpenoids and polyketides that are metabolized in functional prediction and are expressed less in the continuous crop group than in the non-continuous group. This may cause the accumulation of terpenoids in continuous cropping compartments. Terpenoids have been proven to be an important allelopathic substance that causes the obstacle of continuous cropping in plants (Zhang et al., 2014).

## Conclusion

By examining the variation of the continuous cropping microbial community in bulk soil, rhizosphere soil and beetroot, this study provides a systematic understanding of the succession of microbiome composition and environmental factor correlations and their differences in potential function. Continuous cropping changed the soil properties, soil enzyme activity and the growth of sugar beet, affect the community structure and diversity of three compartments bacteria and fungus. Furthermore, continuous cropping significantly affected the fungal community and increased the abundance of potential pathogens. We further found that the three belowground compartments show different structural features of the fungal community from the outside to the inside. Additionally, environmental factors affect fungal microbial communities more intensively than bacteria. A variation in diversity and composition of the microbial community, especially increased relative abundance of potentially pathogenic and changes in bacterial community function could be the main cause of continuous sugar beet cropping obstacle. These findings will further improve our fundamental understanding of continuous cropping plant-microbiome interactions and provide critical new knowledge for future mitigation of continuous cropping obstacles.

## Data availability statement

The datasets presented in this study can be found in online repositories. The names of the repositories and accession numbers can be found below: https://www.ncbi.nlm.nih.gov/bioproject/PRJNA793140, PRJNA793140 (16S data) and https://www.ncbi.nlm.nih.gov/bioproject/PRJNA793139, PRJNA793139 (ITS data).

## Author contributions

RC and GG: conceptualization, methodology, and writing—original draft preparation. GW, YD, and TL: data curation and sample analysis. YW: conceptualization, resources, supervision, and writing—reviewing. LY: investigation and sample analysis. PS: methodology and writing—reviewing. All authors contributed to the article and approved the submitted version.

## Footnotes

1
http://210.22.121.250/CloudPlatform/home
