# Supplementary material for: The response of sugar beet rhizosphere micro-ecological environment to continuous cropping
Source: Front Microbiol. 2022 Sep 7;13:956785. doi: 10.3389/fmicb.2022.956785 (PMC9490479; doi:10.3389/fmicb.2022.956785)
Supplement: Supplementary file 1 [file Table_1.DOCX]

Supplementary Material

# The response of sugar beet rhizosphere micro-ecological environment to continuous cropping

**Rufei Cui^1,3†^, Gui Geng^1,2,3†^, Gang Wang^1,2,3^, Piergiorgio Stevanatod^4^, Yinzhuang Dong^1,2,3^, Tai Li^1,3^, Lihua Yu^1^, Yuguang Wang^1,2,3^***

^1^ National Sugar Crop Improvement Centre, College of Advanced Agriculture and Ecological Environment, Heilongjiang University, Harbin, China

^2^ Heilongjiang Provincial Key Laboratory of Ecological Restoration and Resource Utilization for Cold Region, College of Life Sciences, Heilongjiang University, Harbin, China

^3^ Engineering Research Center of Agricultural Microbiology Technology, Ministry of Education, Heilongjiang University, Harbin, China

^4^ DAFNAE, Dipartimento di Agronomia, Animali, Alimenti, Risorse Naturali e Ambiente, Università degli Studi di Padova, Padova, Italy

**†** These authors have contributed equally to this work and share first authorship

*** Correspondence:**

Yuguang Wang

E-mail: wangyuguang@hlju.edu.cn

**Contents**

**Tables**

Table S1 Microbial richness and a-diversity indexes for different groups.

Table S2 Two-way ANOVA (F) on the diversity of alpha in belowground compartments of different groups of sugar beet.

**Figures**

Figure S1 Morphological change in sugar beet under continuous cropping and non-continuous cropping treatment.

Figure S2 Prediction of metabolic pathways by KEGG level 1 (A), level 2 (B), and level 3 (C).

**Table S1** Microbial richness and a-diversity indexes for different groups.

| Group | Bacteria | | Fungi | |
| --- | --- | --- | --- | --- |
|  | Chao 1 | Richness | Chao 1 | Richness |
| Sc | 5016.3992 ± 190.7999^a^ | 4183.8 ± 250.7607^a^ | 616.6630 ± 33.1386^b^ | 527.0 ± 35.0086^b^ |
| Sn | 5120.2862 ± 186.7452^a^ | 4251.6 ± 273.6308^a^ | 781.8113 ± 103.1900^a^ | 687.4 ± 117.0514^a^ |
| Rc | 4387.9443 ± 311.3261^b^ | 3413.6 ± 294.0739^b^ | 554.1487 ± 22.9918b^c^ | 482.8 ± 30.3934^bc^ |
| Rn | 4613.8911 ± 224.5809^b^ | 3521.2 ± 288.9466^b^ | 533.0580 ± 122.8328^bc^ | 402.4 ± 113.1611^cd^ |
| Bc | 2399.8260± 257.1390^d^ | 1713.8 ± 215.0175^c^ | 397.2700 ± 25.7003^cd^ | 346.4 ± 26.4242^d^ |
| Bn | 2834.2904 ± 133.5383^c^ | 1988.2 ± 95.7108^c^ | 301.3413 ± 63.2291^d^ | 234.2 ± 48.8688^e^ |

Notes:

Sc, continuous cropping bulk soil; Sn, non-continuous cropping bulk soil; Rc, continuous cropping rhizosphere soil; Rn, non-continuous cropping rhizosphere soil; Bc, continuous cropping sugar beet root; Bn, non-continuous cropping sugar beetroot.

Values are means ± standard deviation (n = 5), followed by the same letter for a given factor which are not significantly different (P < 0.05; Wilcoxon test).

**Table S2** Two-way ANOVA (F) on the diversity of alpha in belowground compartments of different groups of sugar beet. Treatments: continuous and non-continuous cropping groups; compartments: bulk soil, rhizosphere soil and beetroot.

|  | Indicators | Treatment | compartment | Treatment * compartment |
| --- | --- | --- | --- | --- |
| Bacteria | Chao 1 | 7.722^*^ | 261.202^**^ | 1.108^NS^ |
|  | Richness | 2.229^NS^ | 193.369^**^ | 0.397^NS^ |
| Fungi | Chao 1 | 0.289^NS^ | 46.076^**^ | 6.773^**^ |
|  | Richness | 0.131^NS^ | 37.984^**^ | 8.399^**^ |

Notes:

* P < 0.05, ** P < 0.01, and ^NS^ showed no significant difference.

**Fig S1** Morphological change in sugar beet under continuous cropping and non-continuous cropping treatment.


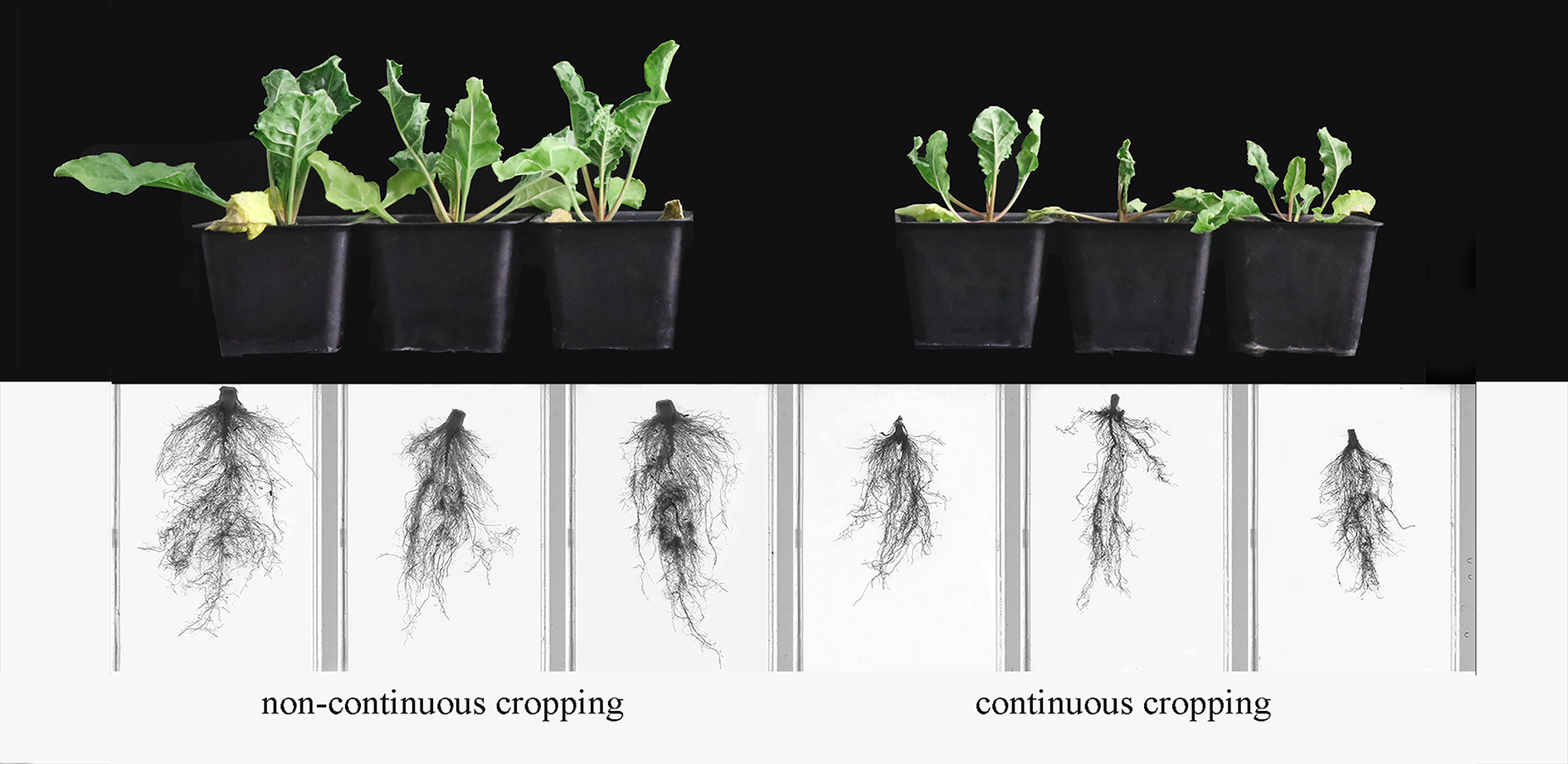


**Fig. S2** Prediction of metabolic pathways by KEGG level 1 (A), level 2 (B), and level 3 (C).**
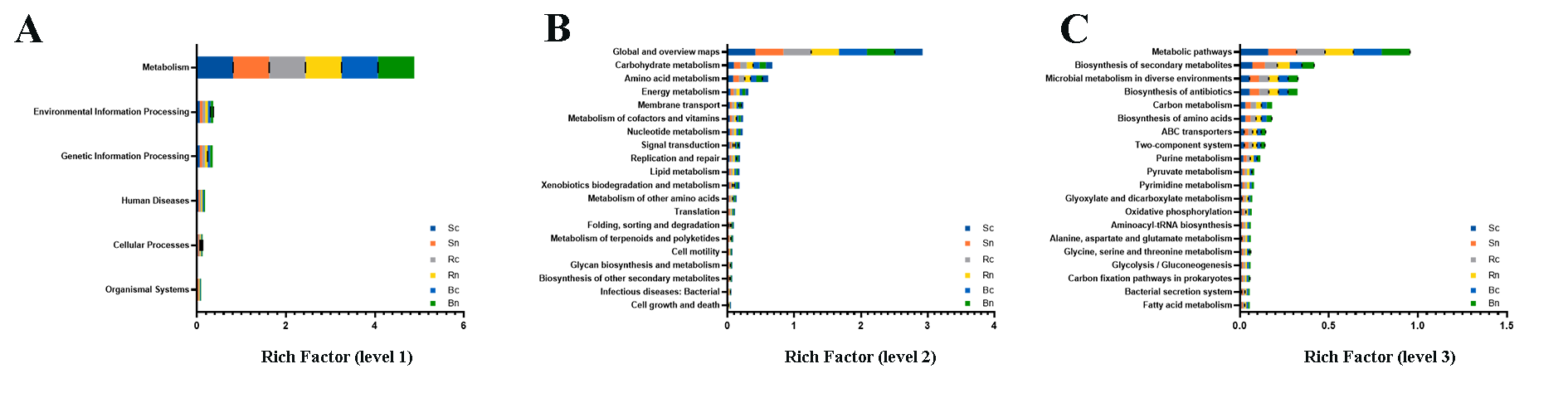
**

Notes:

Sc, continuous cropping bulk soil; Sn, non-continuous cropping bulk soil; Rc, continuous cropping rhizosphere soil; Rn, non-continuous cropping rhizosphere soil; Bc, continuous cropping sugar beetroot; Bn, non-continuous cropping sugar beetroot.
